# Closing the Gap: A Comparison of Engagement Interventions to Achieve Equitable Breast Cancer Screening in Rural Illinois

**DOI:** 10.1089/pop.2021.0382

**Published:** 2022-04-19

**Authors:** Sarah Stewart de Ramirez, Jeremy McGarvey, Abby Lotz, Mackenzie McGee, Tenille Oderwald, Katherine Floess, Roopa Foulger, Melinda Cooling, Jonathan A. Handler

**Affiliations:** ^1^ OSF HealthCare System, Population Health Services, Peoria, Illinois, USA.; ^2^ University of Illinois College of Medicine at Peoria, Department of Emergency Medicine, Peoria, Illinois, USA.; ^3^ OSF HealthCare System, Department of Health Care Analytics, Peoria, Illinois, USA.; ^4^ Beartooth Billings Clinic, Red Lodge, Montana, USA.; ^5^ OSF HealthCare System, OSF OnCall, Peoria, Illinois, USA.; ^6^ OSF Saint Francis Medical Center, Department of Radiation Oncology, Peoria, Illinois, USA.; ^7^ OSF Saint Francis Medical Center, Division of Cancer Support Services, Peoria, Illinois, USA.; ^8^ OSF HealthCare System, OSF Innovation, Peoria, Illinois, USA.; ^9^ Northwestern University Feinberg School of Medicine, Department of Emergency Medicine, Chicago, Illinois, USA.

**Keywords:** breast cancer, population health, health disparities, digital health, patient engagement

## Abstract

Mammography screening rates are typically lower in those with less economic advantage (EA). This study, conducted at an integrated health care system covering a mixed rurality population, assessed the ability of interventions (text messages linking to a Web microsite, digital health care workers, and a community health fair) to affect mammography screening rates and disparity in those rates among different EA populations. Payor type served as a proxy for greater (commercially insured) versus lower (Medicaid insured) EA. 4,342 subjects were included across the preintervention (“Pre”) and postintervention (“Post”) periods. Interventions were prospectively applied to all Medicaid subjects and randomly selected commercial subjects. Applying interventions only to lower EA subjects reversed the screening rate disparity (2.6% Pre vs. −3.7% Post, odds ratio [OR] 2.4 *P* < 0.01). When intervention arms (“Least,” “More,” “Most”) were equally applied, screening rates in both EA groups significantly increased in the More arm (Medicaid OR = 2.04 *P* = 0.04, Commercial OR = 3.08 *P* < 0.01) and Most arm (Medicaid OR 2.57 *P* < 0.01, Commercial OR 2.33 *P* < 0.01), but not in the Least (text-only) arm (Medicaid OR 1.83 *P* = 0.11, Commercial OR 1.72 *P* = 0.09), although this text-only arm was inadequately powered to detect a difference. In summary, targeting interventions to those with lower EA reversed screening rate disparities, text messaging combined with other interventions improved screening rates in both groups, and future research is needed to determine whether interventions can simultaneously improve screening rates for all without worsening the disparity.

## Introduction

Breast cancer is the fourth leading cause of cancer mortality in the United States. Although breast cancer cases have been rising 0.3% per year on average, breast cancer death rates have been falling an average of 1.3% per year from 2010 to 2019.^1^ This decrease can be partially attributed to early detection through mammography screening.^2^ Mathematical models suggest that half of the total reduction can be attributed to mammography screening.^3^ The benefit of mammography requires screening multiple times throughout a woman's lifetime.^4^ For example, the National Comprehensive Cancer Network (NCCN) guidelines recommend annual mammography for most women starting at age 40, and even earlier for women with elevated breast cancer risk.^5^

The Centers for Disease Control and Prevention (CDC) found that 72.8% of all U.S. women reported having a mammogram in 2018.^6^ However, this percentage has been shown to vary greatly across levels of economic advantage (EA). Another study found that the prevalence of mammography screening for women in households with higher incomes had higher rates than those in households with lower incomes.^7^ These disparities have become more pronounced during the COVID-19 pandemic, which dropped screening rates across the country while also widening the disparity gap due to the disproportionate impact of COVID on patients of lower EA.^8^

Previous research has been conducted on breast cancer screening interventions, but little has been reported on the most effective way to reach rural and lower EA populations at high risk for missing their mammography screenings. A published literature review of breast cancer screening interventions concluded that the most effective interventions were simple low-cost actions such as reminder messages and prompts from health care professionals.^9^

However, those studies looked at whether interventions improved breast cancer screening rates as a whole, not whether interventions could be used to reduce disparities among different populations. Community Health Workers (CHW) have been shown to help increase screening rates in vulnerable populations^10^; however, they are difficult to scale over large geographic areas. It is also not known which outreach methods are most effective in closing the disparities across these vulnerable populations.

To help address the knowledge gaps, the authors conducted a prospective interventional study at a multihospital integrated health care system that serves a community of rural and urban patients of differing EA. They sought to determine whether various interventions could reduce screening disparities between women of different EA, and the relative efficacy of interventions in improving screening rates in different EA groups.

## Methods

 The Institutional Review Board approved this study under IRB 1636804-8.

### Inclusion/exclusion

This study was reviewed and approved by the University of Illinois at Peoria IRB. The NCCN clinical practice guideline was utilized to determine study eligibility because it is the guideline used by the health system's clinical service lines.^5^ “Overdue” was defined as all women who were at least 1 year past the date they should have received their last screening, or 2 years in total since the date of their last mammogram. Since the NCCN recommendation is for annual screening starting at 40 years of age, recruitment parameters were set as women 41 years old and older who had not had mammograms in the last 2 years. Forty-one was chosen to allow time for the initial screening to occur after turning 40. Therefore, eligibility was based on the following criteria using data from the health system's data warehouse:

**Inclusion Criteria**
- Female
- Forty-one years old or older at the start of the relevant time frame
- A participant in the hospital system-affiliated accountable care organization with either a selected commercial payor or 1 of 2 selected Medicaid payors
- Aligned with a primary care physician affiliated with the health system
- Has a cell phone number documented in the electronic medical record
- No mammogram in the 2 years before the start of the relevant period

**Exclusion Criteria**
- History of double mastectomy
- From a county with no disparity in breast cancer screening rates
- From a county with less than 5 subjects in an included payor group
- Currently under active treatment for breast cancer

Three intervention arms were designed:

- **Arm 1 (“Least” intervention)**: Subjects received up to 3 text message reminders about the need for a mammogram. Each text message contained a link to a “microsite” (simple website formatted for cell phones) with information about the importance of breast cancer screening and a link to schedule a mammogram online. Subsequent text messages were not sent after the data set showed that a subject had scheduled a mammogram.
- **Arm 2 (“More” intervention)**: Subjects received up to 3 text message reminders about the need for a mammogram. Each message contained a link to a microsite with similar content to Arm 1, but adding slightly more educational content on breast health as well as an opportunity to request support from a digital health worker (DHW) to answer questions and assist with mammogram scheduling. Subjects who scheduled a mammogram received a follow-up text providing positive feedback for having scheduled the mammogram, and no subsequent texts from the study. All subjects who did not explicitly respond “No” to the microsite opportunity to talk with a DHW were contacted by phone by a DHW (up to 3 attempts per subject) to answer questions, assist with scheduling the mammogram as needed, and to offer screening for Social Determinants of Health (SDOH) to further guide assistance.
- **Arm 3 (“Most” intervention)**: Subjects received all interventions in Arm 2, ***plus*** they were also invited to a “Health Fair.” The “Most” designation reflects that this arm included the greatest variety of interventions. At the Fair, breast health trained hospital volunteers provided breast cancer risk reduction education, cancer IQ screening,^11^ wellness screens, SDOH screening, and assistance scheduling or obtaining mammography. The fairs were held on days with availability for walk-in mammography screenings, and so, attendees could get a mammogram that day if desired. If transportation or childcare was needed, volunteers offered assistance during the previsit outreach.

For logistical purposes, the county where the Fair would be held and its 2 sister counties were assigned to Arm 3. Assignment to the other 2 arms was also done at the county level, with counties randomly assigned until a roughly equal number of subjects were assigned to each arm.

Having commercial health insurance (“Commercial group”) served as a proxy for greater EA, and Medicaid insurance (“Medicaid group”) served as a proxy for lower EA since qualification for Medicaid is largely a function of household income.

The study was divided into 2 periods as follows: (1) 5 months before the initiation of interventions, designated the “Pre” period, and (2) 5 months beginning immediately after Pre called the “Post” period. Before the start of Post, subjects meeting the inclusion criteria at that time were prospectively assigned to the Post period group. Near the end of Post, the data set was analyzed to identify those who had met the inclusion criteria at the start of Pre for retrospective assignment to the Pre period group.

For each period, commercially insured subjects were randomly assigned to 1 of 2 subgroups, Intervention Commercial and the Nonintervention Commercial. For both the Pre and Post period, subjects in the Intervention Commercial group and all in the Medicaid group were assigned to an intervention arm based on their home county.

No intervention was applied during the Pre period. During the Post period, Nonintervention Commercial subjects continued to receive no intervention, whereas Medicaid and Intervention Commercial subjects received the intervention appropriate to their study arm.

### Analytical and statistical methodology

All statistical analyses were performed using the R statistical package (version 4.0.0) using a 2-sided alternative hypothesis with a 95% confidence level.

### Primary analysis—disparity impact

The primary outcome studied was disparity in breast cancer screening rates between greater EA (commercially insured) versus lower EA (Medicaid insured) women. For this, a pre/post interventional study was performed. During the Pre period, all in the Commercial group were compared with all in the Medicaid group to determine the mammogram rates and the difference in rates between the groups (the disparity). For the Post period, all arms in aggregate from the Medicaid group were compared with the Nonintervention Commercial group. At the end of Post, the change in each group's screening rate from the Pre to Post period was assessed, and the screening rate disparity between the Nonintervention Commercial group and the Medicaid group was determined.

#### Statistical methodology for the disparity reduction analysis

Generalized estimating equations with an exchangeable correlation structure were used to account for dependencies associated with some subjects being in both the Pre and Post periods. The dependent variable was a binary variable indicating whether or not the subject had a mammogram. Independent variables included insurer group and period (Pre and Post). Patient age, race/ethnicity, and primary language were also included to control for differences in patient demographics between payors.

The interaction between group and period was of particular interest as it would determine if there was a significant difference in the difference between payors (disparity) between periods, or if 1 group showed more change from Pre to Post than the other. *Post hoc* pairwise comparisons of the adjusted mammogram rates were performed using the R package emmeans (version 1.6.1)^12^ to compare adjusted mammogram rates between levels of group within each time period and between time periods within each group.

### Secondary analysis—intervention arm comparisons

A secondary outcome assessed was the effectiveness of intervention arms within each group (ie, within the Intervention Commercial group and within the Medicaid group). In those analyses, for the Pre period, subjects were assigned to an arm based on their home county even though no intervention was done during this period. This allowed an even comparison within arms, since populations and their baseline screening rates differed by county. Although most of these secondary analyses were underpowered, they were performed to identify strong effects or, if none found, to identify trends that might inform future research.

#### Medicaid intervention arm comparisons

To assess and compare the effectiveness of the intervention arms for the Medicaid group, the screening rate for each arm was compared Pre with Post. The screening rates during Post were also compared with one another across arms. All subjects in the Medicaid group were included in both the Pre and Post periods.

#### Commercial intervention arm comparisons

To assess and compare the effectiveness of the intervention arms for the Commercial group, the screening rate for each arm was compared Pre with Post. The screening rates during Post were also compared with one another across arms. Only subjects in the Commercial Intervention group were included in both the Pre and Post periods for this analysis.

#### Statistical methodology for the intervention arm comparisons

In addition to the independent variables of study arm, time period, and the interaction between these variables, county-level mammogram rates during the Pre period were also included as a covariate to control for differences in mammogram rates between the counties that made up the study arms. County-level Pre period rates were calculated for each payor group and included at a subject level. The dependent variable was a binary variable indicating whether or not the subject had a mammogram. Generalized estimating equations with an exchangeable correlation structure were used to account for dependencies associated with some subjects being in both the Pre and Post periods.

Pairwise *post hoc* comparisons of the adjusted mammogram rates were used to compare Pre and Post periods within each intervention arm and between intervention arms within the Pre or Post period, as opposed to comparisons at the reference levels used in the generalized estimating equation. A Bonferroni correction for 3 tests was used to adjust *P*-values for multiple comparisons when comparing study arms within a period. *Post hoc* power analysis was done for the pairwise comparisons to determine if the sample size was adequate to detect a small Cohen's *h* effect size (0.2)^13^ for a 2-proportion *z*-test with unequal sample sizes.

### Descriptive analysis

Descriptive statistics were calculated to characterize the populations and various aspects of program execution, such as response rates after texting.

#### Statistical methodology for the descriptive analysis

Frequencies and proportions were reported for categorical or binary variables, and chi-square or Fisher's exact tests (depending on the minimum expected cell count) were used to compare proportions. Means and standard deviations were reported for continuous variables (such as age in the comparison of demographic variables), and *t*-tests were used when comparing means of continuous variables.

## Results

A total of 4342 women met the criteria for inclusion in the study across both study periods. Within each EA group, the population did not significantly change in age, race/ethnicity, or language between the Pre and Post periods. Comparing the 2 EA groups, the Commercial group was statistically significantly older, more predominantly White/Caucasian, and more likely to speak English as a primary language. However, the 2 groups did not significantly differ in their likelihood to have a cell phone number recorded in the electronic health record (Table 1).

**Table 1.** Patient Demographics

Between time periods						
	Medicaid			Commercial		
	Pre	Post	P	Pre	Post	P
Total *n*	747	859		2836	2539	
Age, mean (SD)	52.2 (7.9)	52.1 (8.0)	0.78	54.0 (7.3)	54.1 (7.4)	0.674
Race/ethnicity, *n* (%)						
White/Caucasian non-Hispanic	645 (86.3)	733 (85.9)	0.825	2636 (92.9)	2351 (92.6)	0.937
African American non-Hispanic	51 (6.8)	65 (7.6)		49 (1.7)	48 (1.9)	
Hispanic/Latino	17 (2.3)	15 (1.8)		49 (1.7)	48 (1.9)	
Other	34 (4.6)	40 (4.7)		102 (3.6)	92 (3.6)	
Language, *n* (%)						
English	721 (96.5)	830 (97.3)	0.662	2806 (98.9)	2510 (98.9)	0.89
Spanish	8 (1.1)	7 (0.8)		13 (0.5)	14 (0.5)	
Other	18 (2.4)	16 (1.9)		17 (0.6)	15 (0.6)	
*Between payors*						
	*Medicaid*		*Commercial*		P	
Total *n*	932		3410			
Age, mean (SD)	51.9 (7.9)		53.8 (7.3)		<0.001	
Race/ethnicity, *n* (%)						
White/Caucasian non-Hispanic	798 (85.6)		3165 (92.8)		<0.001	
African American non-Hispanic	69 (7.4)		61 (1.8)			
Hispanic/Latino	19 (2.0)		64 (1.9)			
Other	46 (4.9)		120 (3.5)			
Language, *n* (%)						
English	902 (96.8)		3374 (98.9)		<0.001	
Spanish	10 (1.1)		16 (0.5)			
Other	20 (2.2)		20 (0.6)			
Cell phone no. in EMR, *n* (%)	853 (91.1)		3105 (89.8)		0.215	

EMR, electronic medical record; SD, standard deviation.

Response metrics related to text messaging were largely similar between the Medicaid and Commercial groups. Repeat messaging (up to 3 in total) did not appear to diminish response rates, as response metrics were often similar or higher for the third text message than the first. Although Medicaid group subjects were more likely to be “no-shows” for their scheduled appointments, no-shows in both groups represented a minority of the scheduled appointments (Table 2). Among those assigned to More and Most interventions, 39% of Commercial and 37% of Medicaid subjects were reached by DHWs, and 29% of the subset of Commercial subjects reached by DHWs and 27% of the subset of Medicaid reached by DHWs consented to the DHW intervention. Those differences were not statistically significant.

**Table 2.** Intervention Response Metrics

	Commercial*, n *(%)	Medicaid*, n *(%)	P
Least interventions			
Message 1			
Total	566	853	
Microsite clicked	68 (12.0)	141 (16.6)	0.023
Response	25 (4.4)	29 (3.4)	0.400
Completed mammogram	24 (4.2)	16 (1.9)	0.013
Opted out	10 (1.8)	13 (1.5)	0.890
Message 2			
Total	532	822	
Microsite clicked	110 (20.4)	196 (23.8)	0.150
Response	42 (7.7)	61 (7.4)	0.949
Completed mammogram	15 (2.8)	8 (1.0)	0.019
Opted out	5 (0.9)	1 (0.1)	0.037
Message 3			
Total	490	774	
Microsite clicked	101 (20.6)	181 (23.4)	0.278
Response	46 (9.4)	66 (8.5)	0.672
Completed mammogram	19 (3.9)	24 (3.1)	0.560
Opted out	3 (0.6)	2 (0.3)	0.382
Most interventions			
Total	175	287	
Response: “Learn more & RSVP”	5 (2.9)	13 (4.3)	0.514
Health fair date	1 (0.6)	4 (1.4)	0.650
Total completed mammograms	36 (20.6)	41 (14.3)	0.103
Patient-level appointment metrics			
Total	566	853	
No appointment scheduled	453 (80.0)	709 (83.1)	0.160
Schedule appointment w/o mammogram	23 (4.1)	54 (6.3)	0.084
Schedule appointment w/mammogram	90 (15.9)	90 (10.6)	0.004
Appointment-level metrics			
Total	136	186	
Canceled	18 (13.2)	27 (14.5)	0.87
No show	5 (3.7)	31 (16.7)	0.001
Rescheduled	23 (16.9)	38 (20.4)	0.515
Mammogram completed	90 (66.2)	90 (48.4)	0.002

Responses to the health fair invitation were very low and did not significantly differ between the EA groups (Table 2). None of those offered SDOH screening and possible assistance accepted the offer.

**FIG. 1.**
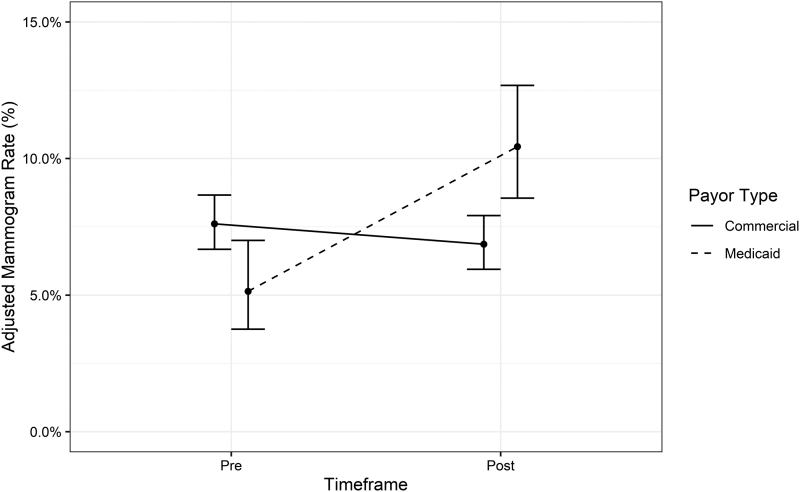
Mammography rates in commercially insured compared with Medicaid-insured patients, before and after interventions, were applied to the Medicaid-insured patients. Rates adjusted for age, race/ethnicity, and primary language.

The primary analysis assessed the ability for targeted intervention (intervening on Medicaid subjects only) to affect disparity in breast cancer screening rates between EA groups (Fig. 1). During the Pre period, 8.0% of the Commercial group and only 5.4% of the Medicaid group received a mammogram (odds ratio [OR] = 0.66, *P* = 0.02). No one during Pre received an intervention. Mammogram screening rates for the Nonintervention Commercial group during Post did not significantly differ from the rates for the Commercial group during the Pre period (Post 7.3%, *n* = 2539 vs. Pre 8.0%, *n* = 2836; OR = 0.90, *P* = 0.28).

On the contrary, for Medicaid patients during the Post period (when all Medicaid subjects were assigned to an intervention arm), the mammogram screening rate was significantly higher than during the Pre period (Post 11.0%, *n* = 853 vs. Pre group 5.4%, *n* = 747; OR = 2.15, *P* < 0.001). Applying the interventions to those with Medicaid while withholding them from those with commercial insurance significantly reduced, and in fact reversed the absolute disparity in screening rates between the 2 groups (Pre 2.6% disparity in favor of the Commercial group versus Post 3.7% disparity in favor of the Medicaid group, OR = 2.40, *P* < 0.001).

In the Medicaid Intervention Arm Comparison, Pre versus Post mammography rates were compared within each intervention arm, and Post period mammography rates were compared across arms (Table 3). For the “Least” intervention arm, the rate did not significantly change from that of the Pre period (although the study was underpowered to detect this difference), whereas for both the “More” and “Most” intervention arms, the rates were significantly higher in the Post than the Pre period. Looking only at the Post period, the different intervention arms did not significantly differ from one another in mammography rates; however, the study was inadequately powered to detect a difference in these comparisons.

**Table 3.** Pairwise Comparisons of Adjusted Mammogram Rates for Medicaid Patients

Within study arm							
Group	n	Comparison	n	OR	95% CI		P
Pre-Least interventions	244	Post-Least interventions	283	1.83	0.88	3.8	0.106
Pre-More interventions	241	Post-More interventions	283	2.04	1.03	4.05	0.042
Pre-Most interventions	262	Post-Most interventions	287	2.57	1.42	4.66	0.002
*Between study arms*							
*Group*	n	*Comparison*	n	*OR*	*95% CI*		*Adj.* P
Pre-More interventions	241	Pre-Least interventions	244	1.03	0.37	2.83	1
Pre-Most interventions	262	Pre-Least interventions	244	1.22	0.46	3.2	1
Pre-Most interventions	262	Pre-More interventions	241	1.18	0.47	3	1
Post-More interventions	283	Post-Least interventions	283	1.15	0.55	2.37	1
Post-Most interventions	287	Post-Least interventions	283	1.71	0.88	3.34	0.162
Post-Most interventions	287	Post-More interventions	283	1.49	0.79	2.81	0.385

Adj. *P*, adjusted *P*-value; CI, confidence interval; OR, odds ratio.

In the Commercial Intervention Arm Comparison (Table 4), the “Least” intervention arm mammography rate did not significantly change from that of the Pre period, although there was a trend toward significance (OR = 1.72, *P* = 0.09) and the study was underpowered to detect a difference. For both the “More” and “Most” intervention arms, the rates were significantly higher in the Post than the Pre period. Looking only at the Post period, the different intervention arms did not significantly differ from one another in mammography rates, however, the study was inadequately powered to detect this difference.

**Table 4.** Pairwise Comparisons of Adjusted Mammogram Rates for Commercial Patients

Within study arm							
Group	n	Comparison	n	OR	95% CI		P
Pre-Least interventions	173	Post-Least interventions	185	1.72	0.92	3.22	0.087
Pre-More interventions	187	Post-More interventions	206	3.08	1.47	6.46	0.003
Pre-Most interventions	167	Post-Most interventions	175	2.33	1.29	4.23	0.005
*Between study arms*							
*Group*	n	*Comparison*	n	*OR*	*95% CI*		*Adj.* P
Pre-More interventions	187	Pre-Least interventions	173	0.76	0.26	2.24	1
Pre-Most interventions	167	Pre-Least interventions	173	1.07	0.45	2.54	1
Pre-Most interventions	167	Pre-More interventions	187	1.4	0.48	4.09	1
Post-More interventions	206	Post-Least interventions	185	1.36	0.61	3.06	1
Post-Most interventions	175	Post-Least interventions	185	1.44	0.74	2.83	0.574
Post-Most interventions	175	Post-More interventions	206	1.06	0.5	2.24	1

Adj. *P*, adjusted *P*-value; CI, confidence interval; OR, odds ratio.

**FIG. 2.**
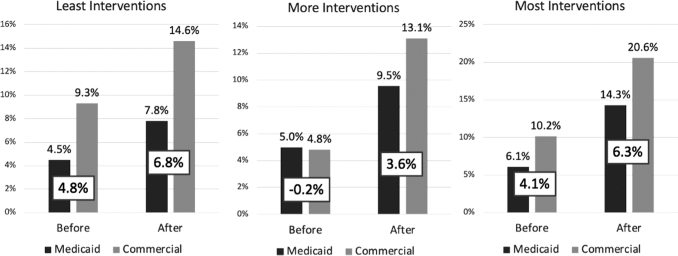
Mammography rates and disparity between commercially insured and Medicaid-insured patients by intervention and time frame.

Analysis of the interventions' impact on likely first-time mammography rates (women ages 41–42) similarly revealed a significant increase in rates for lower EA women (OR = 2.79, *P* = 0.047). The presumed rates did not change significantly for high EA women (OR = 4.04, *P* = 0.151), but this comparison was inadequately powered. When comparing the impact of each arm on screening rate disparity (Fig. 2), the absolute disparity increased in each arm after the intervention, although this was not analyzed for significance.

## Discussion

Lower EA populations have been shown to have a greater likelihood of noncompliance with cancer screening guidelines than their higher EA counterparts.^7^ Consistent with these findings, the authors found that lower EA subjects had a 17% lower breast cancer screening rate (relative difference) than their higher EA counterparts before intervention. However, interventions targeted to lower EA subjects were able to eliminate and even reverse that disparity.

Deavenport et al reported that, among low-income Hispanic women in community health clinics, a theory-based intervention led to stronger intentions to get screening mammograms compared with a control group.^14^ Cardarelli et al demonstrated that women who attended 8 weekly sessions of an educational program were more than 10 times more likely than controls to receive a screening mammogram during follow-up.^15^ This study builds on these findings by demonstrating the ability of targeted interventions to affect the real-world disparity in screening rates between more versus less economically advantaged women (Fig. 2).

During the planning phases of this study, it was unknown whether those with low EA would be less likely to have cell phones or less able to access the mobile phone-optimized microsites. No evidence of a difference between the lower and higher EA groups in either of these measures was found (Table 1).

There was a significantly higher rate of “no-shows” to scheduled mammography appointments in the lower EA group, suggesting that appointment reminders offering a simple mechanism to reschedule if needed, and perhaps other interventions to reduce no-shows, may be especially important in that group.

As a secondary study outcome, the authors assessed intervention arms within a payor group to identify which arms significantly improved screening rates. The Least intervention arm (SMS texting only) did not reach statistical significance for either group (Tables 3 and 4), although a trend (*P* = 0.087) toward significance was seen for the Commercial group (Table 4). These trends suggest that further study is warranted to determine if interventions beyond simple text notifications alone are required to get the desired response.

In both payor groups (Medicaid and Commercial), the screening rates significantly increased for both the More and Most intervention arms (Tables 3 and 4). Nearly 40% of all subjects in those groups were reached by a DHW, and about 28% of those reached consented to a discussion, during which the DHW answered questions, assisted with scheduling mammograms, and offered to help identify and address SDOH. Of those activities, the authors only tracked who chose to take the SDOH survey. Even though none of the subjects opted for the survey, many DHWs anecdotally reported they felt the most frequently helpful information they provided when answering questions related to insurance coverage for the mammograms, especially for the Medicaid subjects.

The authors also compared intervention arms within a payor group to identify whether any study arm was significantly different from another in effectiveness. For both the Commercial and Medicaid groups, no study arm proved statistically significantly more effective than the others. Since the analysis of this outcome was underpowered, no firm conclusions can be drawn, but the analysis identifies some areas of interest for future study, described in the next section.

Others have demonstrated the utility of text message reminders for women with existing mammography appointments, with at least 1 noting particular value in a rural or hard-to-reach population.^16,17^ Reminders among those not yet scheduled but due or overdue for a mammogram have also been found useful in studies using modalities such as mailed letters, automated telephone calls, and manual phone calls, especially if individualized and relevant to the patient's specific needs.^18,19^

Lakkis et al studied the effectiveness of more informative versus briefer text messages in encouraging mammogram scheduling for women who were more than 2 years overdue for screening, finding both equally effective.^20^ Others compared communication modalities with one another, such as Richardson et al's comparison of mailed reminders to telephone reminders for New Zealand women for breast cancer screening, finding no difference between them.^21^ Lee et al demonstrated that a “mobile phone app-based intervention combined with health navigator service was a feasible, acceptable, and effective intervention mechanism to promote breast cancer screening in Korean American immigrant women.”^22^

Similarly, this study found that a combination of mobile phone-based intervention plus a DHW service (with or without a health fair invitation) can improve breast cancer screening rates, including among women of low EA newly eligible for their screening mammogram (intervention impact among newly eligible women of higher EA is uncertain). This work also adds new information regarding the ability of interventions to reduce screening disparities among those of different EA, the effectiveness of each intervention within populations of different EA, comparisons between multiple intervention arms within a payor group, and the inclusion of a health fair as one of the interventions to improve screening rates.

The More and Most intervention groups differed only by the inclusion of an invitation to the Health Fair in the Most group. However, because the subjects in the Most group were from different counties than those in the More group, and the Most group had a different baseline mammography rate and included a tertiary care center unlike the other studied counties, the impact of the Fair on screening rates could not be cleanly assessed. Although the highest screening rates in this study were achieved in the group that received the Health Fair invitation, the actual response rates to the Health Fair invitations were very low.

Although the authors attempted to control for differences in baseline screening rates between arms, the (not statistically significantly) higher rates in the Most group could be due to a baseline greater ability of the other (non-Fair) interventions to impact screening rates in this group. Alternatively, perhaps merely offering an invite to the Fair was enough to stimulate some to obtain a mammogram. Finally, the Health Fair was open to all and attendance was not mapped to participation in the study, and so, some who did not respond to the invitation may have participated in and benefited from the Health Fair. For these reasons, additional research is warranted to assess the benefit of a Health Fair intervention.

This study focused on disparities related to economic status as opposed to disparities related to race and ethnicity because an internal analysis of the health care system data for the hospital system utilized in this study suggested that lower EA (using health insurance payor as a proxy) may be a more significant driver of screening rate disparities among women than race, at least in the predominantly White, mixed rurality, mixed EA population served by this system. However, studies have also indicated that disparities exist among breast cancer screening for minority and immigrant women.^23^ Future research might assess these interventions in an even more demographically diverse population.

This study demonstrates that certain interventions can improve screening rates in women of greater EA. However, the disparity trends seen when interventions were applied equally to all highlight the importance of further study to determine if such a strategy might lead to even greater disparity favoring those with greater EA. These results could guide future researchers when designing a follow-up trial to determine whether an interventional strategy exists that benefits all women while also reducing screening rate disparities.

## Limitations and Future Study

This study had several limitations. Since all of the subjects were patients of a particular health system, it was assumed that subjects obtained their mammograms from that system. Mammograms obtained from an outside institution were not captured, potentially affecting the results. Likewise, it was assumed that all providers were using the NCCN guidelines in the context of shared decision-making because that is the health system's policy. If providers were utilizing the American Cancer Society or the U.S. Preventive Services Task Force guidelines that recommend mammography less frequently than NCCN, the intervention messaging could have been promoting screening to women the clinicians had not targeted.^24,25^

The authors assumed that commercially insured subjects had greater EA than Medicaid-insured subjects. However, individual factors may influence financial stability in subjects who are commercially insured, and some subjects may qualify for Medicaid for reasons other than income threshold alone. It was also assumed that commercial subjects were homogenous in economic profile; however, those with commercial insurance vary in their level of EA.

Rural populations with commercial insurance who work in the service and retail industry may be especially vulnerable due to the irregularity of their work. SDOH screening would have identified the subgroup of vulnerable commercial patients with greater EA; however, the subjects offered SDOH screening elected not to participate. Additional efforts to address mammography barriers included offering transportation and childcare services to subjects in the “Most” arm who attended the Health Fair; however, no participants utilized these services. Future research is needed to understand how best to elicit these needs, address them, and understand if the groups who utilize these resources are significantly different from others in the same payor group.

The interventions in this study were grouped into categories, and there were multiple differences between the Least and the More interventions, obfuscating which change or changes were responsible for the impact on screening rates. This is an area for future study.

This study was planned before, but conducted during, the global COVID-19 pandemic. This may have affected the results of the study, as the pandemic may have affected subjects' decisions to obtain a mammogram. Nationally, cancer screening has been negatively affected by the pandemic. Breast cancer screening tests received by women through a CDC Program declined by 87% throughout 2020.^26^ A follow-up study at a time when willingness to receive care (and the ability to access it) is less affected by a pandemic could prove fruitful.

## Conclusion

Interventions can increase breast cancer screening above baseline rates from usual primary care, and targeting the interventions to specific groups can address health care disparities. Accomplishing both of those goals at the same time, however, may be more challenging. Future research informed by this work will enhance understanding of how best to leverage new digital tools alongside traditional engagement strategies in the service of optimizing population health and reducing health disparities.
